# Co‐Developing an Indigenous‐centred Model of Dementia Care in Alberta, Canada: a qualitative study

**DOI:** 10.1002/alz70860_098927

**Published:** 2025-12-23

**Authors:** Jaiden Kuchinka, Lisa Zaretsky, Meagan Ody, Lisa Bourque Bearskin, David B. Hogan, Jayna Holroyd‐Leduc, Dallas Seitz, Zahra Goodarzi, Vivian Ewa, Richard T. Oster, Shannon Ruzycki, Jennifer D Walker, Pamela Roach

**Affiliations:** ^1^ University of Calgary, Calgary, AB, Canada; ^2^ University of Victoria, Victoria, BC, Canada; ^3^ Hotchkiss Brain Institute, University of Calgary, Calgary, AB, Canada; ^4^ Alberta Health Services, Edmonton, AB, Canada; ^5^ McMaster University, Hamilton, ON, Canada

## Abstract

**Background:**

Current evidence favours a shift away from pharmacological to non‐pharmacological approaches for the prevention and management of non‐cognitive symptoms of dementia. With the limited effectiveness of pharmacological treatments and their risk of adverse effects, it is imperative to understand the effectiveness of non‐pharmacological approaches to dementia care and lived experience. However, there has been little work to date to adapt non‐pharmacological approaches in partnership with Indigenous people. The current model of dementia care is unsafe and inadequate and continued inaction will only lead to continued harm. The primary aim of this research was to develop an implementation‐ready approach to Indigenous‐centered dementia care that has been co‐designed with partnering Indigenous communities.

**Method:**

This study prioritized ethical engagement with Indigenous community members. Participants were recruited through community social media and newsletters. Data were co‐analysed using Indigenous approaches to thematic qualitative analysis to draft a preliminary structure for the Indigenous‐centred dementia care approach. A modified Nominal Group Technique was held with a group of health care providers and decision makers that led to agreement on content and design, as well as the development of training materials for facilitators.

**Result:**

Four focus groups (five Indigenous participants with experience of dementia/group) were completed between October and December 2024 for participants to discuss potential content and modes of delivery of an Indigenous‐centred dementia care intervention. Participants identified the need for: 1) culturally‐specific brain health education that includes information on what dementia is; 2) Indigenous‐specific risk identification and dementia prevention; 3) dementia‐friendly Indigenous cultural and spiritual ceremonies; 4) programs for community and facility‐living seniors that involved the traditions of local Indigenous Nations (e.g. jigging, beading, etc); and 5) in‐depth training for staff to deliver the programming.

**Conclusion:**

This work will impact numerous populations that need culturally specific approaches to dementia care policy and planning by informing an ethical process to engaging with diverse populations. This work can help health and continuing care services provide community‐specific approaches to non‐pharmacological dementia care.

